# Supplementary figures and images for: Whole-Genome DNA Methylation Profiling of CD14+ Monocytes Reveals Disease Status and Activity Differences in Crohn’s Disease Patients
Source: J Clin Med. 2020 Apr 8;9(4):1055. doi: 10.3390/jcm9041055 (PMC7230341; doi:10.3390/jcm9041055)

# CD vs non-CD DMR 1

chr7:51538650-51539678

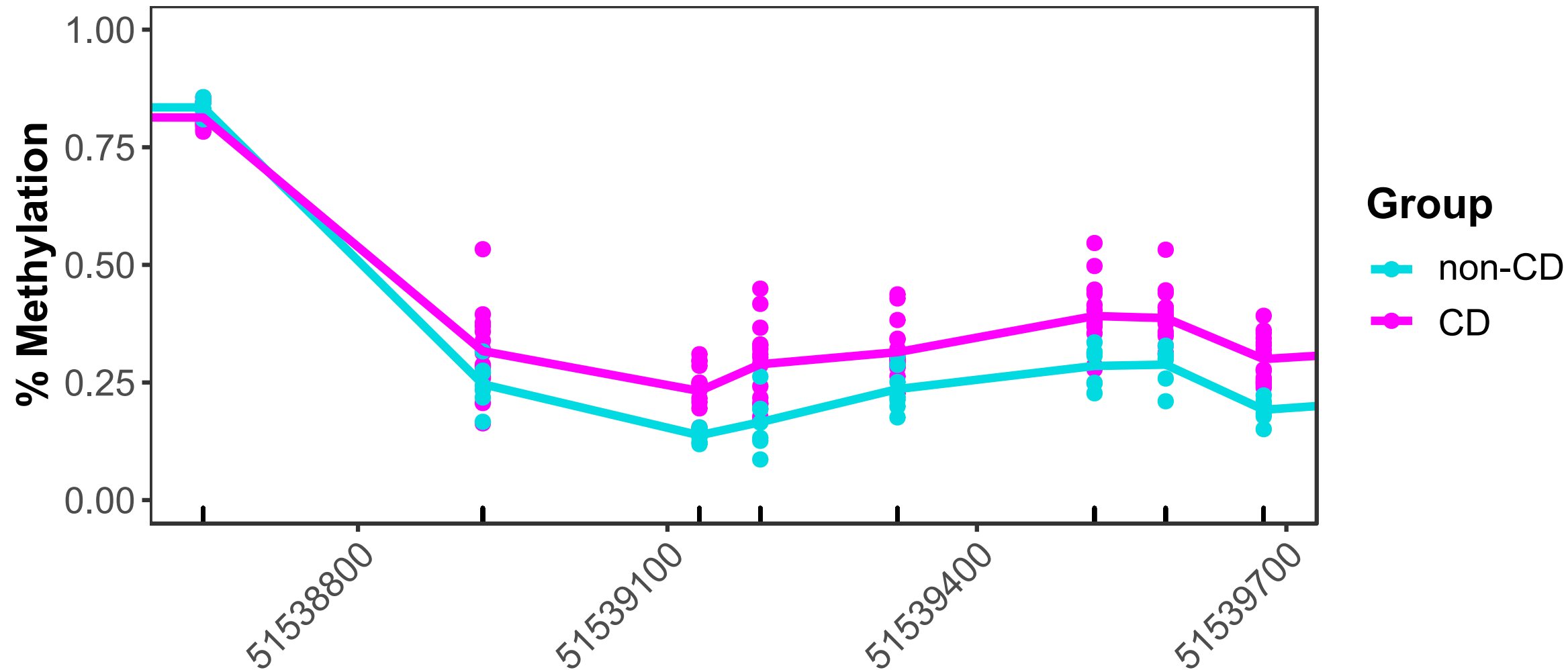

Supplement: Supplementary file 1 [file jcm-09-01055-s001.zip › supplementary/figS1.pdf]
